# Development and Psychometric Evaluation of the Japanese Version of the Physical Effort Scale

**DOI:** 10.2188/jea.JE20250530

**Published:** 2026-10-05

**Authors:** Keishi Soga, Hiroyuki Sasai, Yasuyuki Taki, Boris Cheval, Keita Kamijo

**Affiliations:** 1 Smart-Aging Research Center, Institute of Development, Aging and Cancer, Tohoku University, Sendai, Japan; 2 Research Team for Promoting Independence and Mental Health, Tokyo Metropolitan Institute for Geriatrics and Gerontology, Tokyo, Japan; 3 Department of Aging Research and Geriatric Medicine, Institute of Development, Aging and Cancer, Tohoku University, Sendai, Japan; 4 Univ Rennes, École normale supérieure de Rennes, VIPS^2^, Rennes, France; 5 Faculty of Liberal Arts and Sciences, Chukyo University, Nagoya, Japan

**Keywords:** physical activity, exercise, Theory of Effort Minimization in Physical Activity (TEMPA), cross-cultural research

## Abstract

**Background:**

The Physical Effort Scale (PES) assesses tendencies to approach or avoid physical effort and provides a framework for examining how automatic effort-avoidance tendencies influence physical activity (PA) behavior. To facilitate cross-cultural PA research, this study developed and validated a Japanese version of the PES (PES-JP) following cross-cultural adaptation guidelines.

**Methods:**

An online survey was conducted among 398 Japanese aged 20–59 years.

**Results:**

Exploratory analyses suggested a two-factor structure of the PES-JP (approach and avoidance), and both subscales demonstrated high internal consistency (α = 0.92 and 0.87, respectively). Concurrent validity was supported by associations with PA. The approach score was positively related to moderate-to-vigorous PA (MVPA; β = 0.19) and negatively to sitting time (β = −0.17). In contrast, the avoidance score showed the opposite pattern (β = −0.13 for MVPA, β = 0.20 for sitting). The global score (approach minus avoidance), which reflects the overall tendency to approach rather than avoid physical effort, showed a similar pattern to the approach dimension (β = 0.19 for MVPA, β = −0.22 for sitting). Test-retest reliability was acceptable (intraclass correlations, 0.65–0.81).

**Conclusion:**

The PES-JP demonstrates sound psychometric properties for assessing tendencies toward physical effort in Japanese adults and provides a useful tool for research on individual differences in PA behavior. The scale may also facilitate cross-cultural investigations of how tendencies toward physical effort influence PA across diverse populations.

## INTRODUCTION

Physical inactivity has long been recognized as a global pandemic.^1^ It is responsible for approximately 6 to 10 percent of major non-communicable diseases, such as coronary heart disease, type 2 diabetes, and breast and colon cancer, and accounts for about 9 percent of all deaths worldwide.^2^ Even without knowing such specific figures, the idea that physical activity (PA) is beneficial for health is now widely accepted as common knowledge. Yet, despite widespread recognition of the benefits of PA, physical inactivity remains a global pandemic. Understanding why this pandemic persists therefore remains a critical issue.

Several theoretical models have been proposed to explain engagement in health-related behaviors, including PA. Many sociocognitive frameworks emphasize reflective and self-regulatory processes, such as planning and self-efficacy, as key determinants of behavior.^3^^–^^5^ While these approaches have substantially advanced understanding of PA engagement, they primarily address conscious regulation and place relatively less emphasis on automatic processes that may constrain or facilitate PA behavior. The Theory of Effort Minimization in Physical Activity (TEMPA), proposed by Cheval and Boisgontier,^6^ offers a complementary perspective. According to TEMPA, humans possess a fundamental tendency to minimize physical effort, rooted in evolutionary and neurophysiological constraints. This tendency is thought to operate largely automatically, with brain systems prioritizing energy-efficient actions by default. Such processes can undermine effortful behavior by systematically favoring energy-efficient actions, thereby constraining engagement in PA. For example, when individuals are presented with a choice between stairs and escalators, the vast majority tend to select the escalator, highlighting a robust preference for energy-efficient options in everyday contexts.^7^ These everyday choices can be viewed as examples of how automatic effort-minimization processes shape behavior more broadly. From this perspective, limited engagement in PA may reflect not only reflective self-regulatory factors, such as planning and self-efficacy, but also an automatic predisposition to conserve energy.

Beyond proposing a general tendency toward effort minimization, TEMPA highlights substantial interindividual variability: some people are less drawn to effort-free options and more inclined to engage in effortful behaviors.^6^^,^^8^ Consistent with this view, previous studies have also reported considerable differences in how strongly people are inclined to avoid physical effort.^9^^,^^10^ Such individual differences may help explain why some people are more likely than others to engage in physically demanding activities in daily life. The Physical Effort Scale (PES) was developed to empirically capture such individual differences and to examine how approach and avoidance tendencies toward physical effort relate to PA behavior.^11^ The PES is an eight-item self-report questionnaire comprising four items that measure approach tendencies and four items that capture avoidance tendencies toward physical effort. The scale has demonstrated good internal consistency, test-retest reliability, and construct validity. Notably, the PES has shown concurrent validity through its associations with self-reported PA levels assessed by the International Physical Activity Questionnaire - Short Form (IPAQ-SF),^12^ demonstrating that individuals with higher approach and lower avoidance tendencies tend to engage in more moderate-to-vigorous PA (MVPA). Importantly, the PES has also demonstrated strong psychometric validity in both English^11^ and French^13^ versions, underscoring its cross-linguistic applicability.

As physical inactivity remains a global public health issue, it is crucial to better understand the factors that shape engagement in PA across populations. In particular, how people from different cultural backgrounds perceive and evaluate physical effort may play an important role in influencing PA behavior. The development of culturally adapted measurement tools is vital, not only to advance cross-national comparative research, but also to identify individual differences in the subjective effort experienced during physical activity, which may help explain variability in intervention responsiveness beyond established self-regulatory or affective measures. Accordingly, the present study aimed to develop and validate a Japanese version of the PES (PES-JP), thereby enabling cross-cultural investigations into how tendencies toward physical effort influence PA behavior.

## METHODS

### Scale translation

The translation process followed the guidelines of Wild et al.^14^ The procedural flow of the scale translation is illustrated in Figure 1, and supplemental methods are provided in eMaterial 1, eMaterial 2, eMaterial 3, eMaterial 4, and eMaterial 5. Two Japanese researchers independently produced forward translations, which were harmonized through discussion with a third author. A professional translator then performed back-translation, and both the Japanese authors and the original scale developer confirmed conceptual equivalence. Cognitive debriefing was subsequently conducted with Japanese students, and minor revisions were made to improve clarity. A preliminary validation study with 200 participants compared two versions of one negatively worded item (ie, Item 5); the version with explanatory labels demonstrated stronger validity (eMaterial 2). Accordingly, this version was adopted as the final PES-JP (eMaterial 5). A full description of the translation process is provided in eMaterial 1.

**Figure 1.**
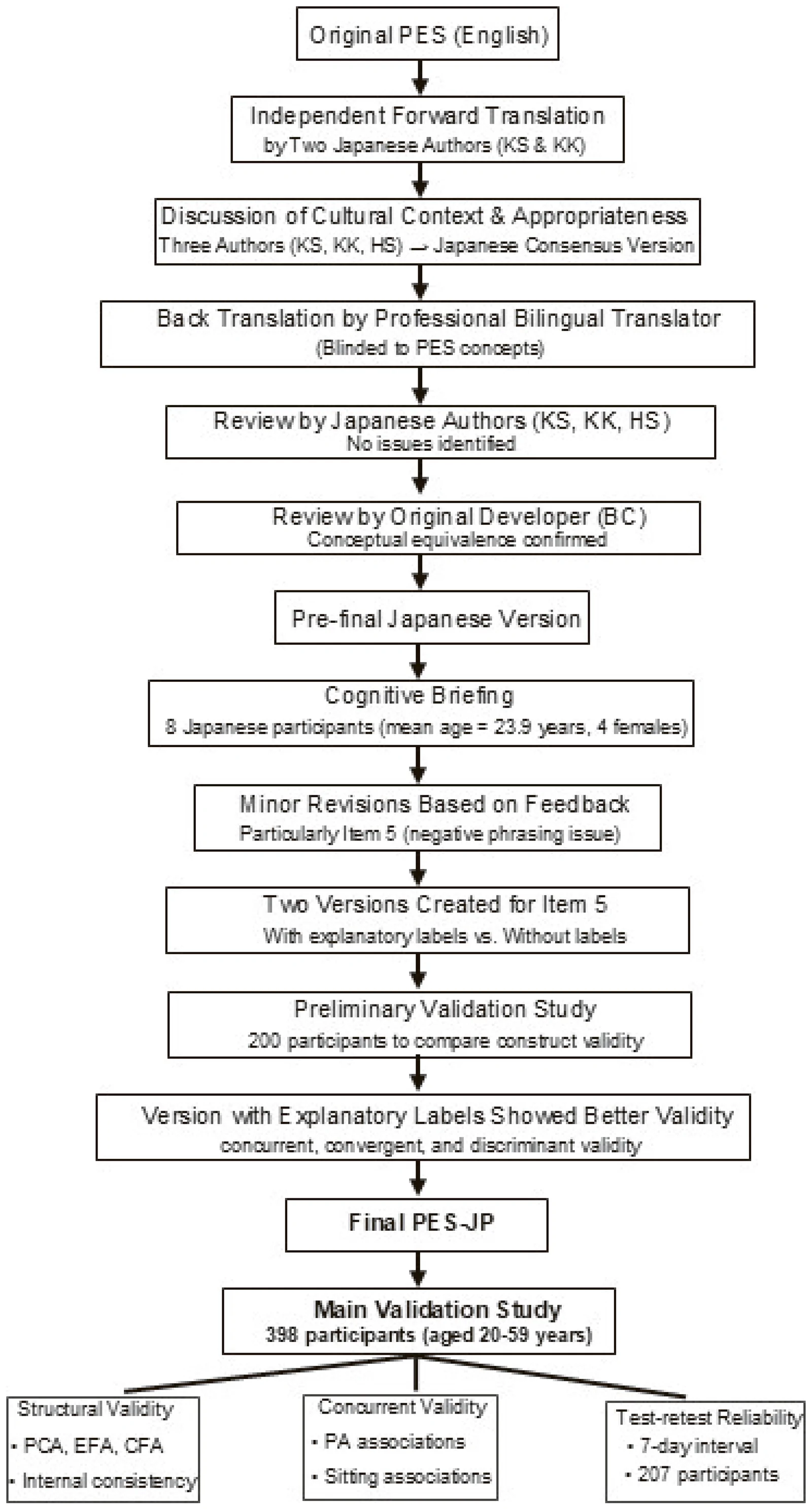
Translation and validation process of the PES-JP. CFA, confirmatory factor analysis using the lavaan package; EFA, exploratory factor analysis; PA, physical activity; PCA, principal component analysis; PES, Physical Effort Scale; PES-JP, Japanese version of the PES.

### Procedure

This study was approved by the Human Research Ethics Committee of Chukyo University (2024-016) and the Ethics Committee of the Graduate School of Medicine, Tohoku University (2025-1-190). The main survey was conducted online. The entire web-based survey process, including participant recruitment and data collection, was conducted by Cross Marketing Inc., a leading survey company in Japan with a panel of over 10 million registered monitors.^15^ Data collection was conducted in April and May 2025. A total of 400 participants were recruited for this study to ensure sufficient representation across different age groups and sexes. This sample size was determined based on commonly accepted recommendations for validation studies, which suggest recruiting at least 10 participants per item when evaluating the structure and reliability of a scale.^16^ Additionally, while the original validation study of the PES by Cheval et al^11^ (study 3) included 297 undergraduate students, we selected a larger and more diverse sample spanning ages 20–59 years to enhance the generalizability of the findings. Prior to accessing the questionnaires, all participants were given a detailed explanation of the study and provided their informed consent electronically. To be eligible for the study, participants had to meet the following criteria: 1) be native Japanese speakers; 2) be able to engage in PA without any medical contraindications; and 3) be aged from 20 through 59 years. Further details on recruitment procedures and stratification are provided in eMaterial 1.

### Measures

The PES-JP consists of eight items (four approach, four avoidance) rated on a 5-point Likert scale, from which a global score (Approach − Avoidance) was calculated. Concurrent validity was examined with PA levels (IPAQ-SF^17^). For comparison with the original and French versions of the PES,^11^^,^^13^ we additionally explored associations between PES-JP scores and theoretically related constructs (eg, motivation, automaticity, attitudes, and intention toward PA). As validated Japanese versions of most of these questionnaires were not available, these measures were translated by the authors and are reported for reference purposes only in the supplemental materials (eMaterial 1 and eMaterial 3), rather than being used as primary evidence of validity. Test-retest reliability was assessed in a subsample after a 7-day interval. Details of the measures and scoring procedures are provided in eMaterial 1.

### Statistical analysis

All statistical analyses were performed using R software (version 4.4.1; R Foundation for Statistical Computing, Vienna, Austria). The structural validity of the PES-JP was examined using a series of analytical approaches. First, principal component analysis was employed to determine the potential number of underlying components. Subsequently, exploratory factor analysis with promax rotation was conducted to identify the latent variables associated with each item in the PES-JP. Finally, confirmatory factor analysis was performed to verify the proposed two-factor structure of the scale. The goodness of fit of the hypothesized two-dimensional model was evaluated using multiple indices: the Comparative Fit Index (CFI), Tucker-Lewis Index (TLI), Root Mean Square Error of Approximation (RMSEA), and Standardized Root Mean Square Residual (SRMR). The following criteria were used to indicate acceptable model fit: CFI and TLI values greater than 0.95, RMSEA less than 0.06, and SRMR less than 0.08.^18^^,^^19^

Following the methodology of the original study,^11^ the concurrent validity was evaluated by examining the association between PES-JP scores and self-reported measures of habitual PA, including moderate-intensity PA, vigorous-intensity PA, and MVPA, as well as time spent walking and sitting. To further assess the explanatory utility of the scale, hierarchical regression analyses were conducted. These analyses aimed to determine whether physical effort approach and avoidance tendencies, and the global score, as measured by the PES-JP, explained additional variance in MVPA levels, after adjusting for demographic factors (age and sex). In subsequent models, each of the following PES-JP scores was added separately to the initial model: a) physical effort approach tendencies, b) physical effort avoidance tendencies, and c) global score. This hierarchical approach allowed us to examine the unique contribution of each PES-JP dimension in explaining variance in the dependent variable, independent of the effects of demographic factors.

Finally, the temporal stability of the PES-JP was re-examined in a subsample of participants, with approximately half of the initial respondents completing the PES a second time. This analysis employed two complementary methods to comprehensively assess test-retest reliability: weighted Kappa coefficients were calculated to assess the stability of individual items at the ordinal level, accounting for the ordered nature of the 5-point Likert scale responses, while intraclass correlation coefficients (ICC) were used to evaluate the absolute agreement and consistency of continuous total scores across the two time points. Test-retest reliability was evaluated based on contemporary criteria. Weighted Kappa coefficients were interpreted according to McHugh,^20^ with values ≥0.40 indicate weak to strong agreement. ICC values were interpreted using the guidelines by Koo and Li,^21^ where values ≥0.50 indicate moderate-to-excellent reliability.

## RESULTS

### Participants

According to the IPAQ scoring criteria,^22^ two participants reporting a total PA time exceeding 960 minutes per day was excluded from the analysis. A total of 398 Japanese participants were included in the main analyses, with 50% identifying as female. The mean age of participants was 40.2 years (standard deviation [SD], 11.1; range, 20–59 years). Participants reported 1,421.7 (SD, 2,174.9) MET-minutes per week of MVPA. The mean PES-JP approach score was 2.7 (SD, 1.0), and the mean avoidance score was 3.1 (SD, 1.0) on the 5-point Likert scale. For the temporal stability analysis, 207 participants from the initial sample completed the PES-JP twice with a 7-day interval. The mean age was 40.0 (SD, 11.2) years, with 46.4% identifying as female.

### Structural validity

Principal component analysis of the 8-item PES-JP suggested a two-component solution, with the first two components explaining 77.2% of the variance. Exploratory factor analysis with promax rotation confirmed the expected two-factor structure (χ^2^ = 46.19, df = 13, *P* < 0.001). All approach items loaded on Factor 1 with loadings ranging from 0.81 to 0.91, while all avoidance items loaded on Factor 2 with loadings ranging from 0.65 to 0.94. Confirmatory factor analysis was conducted to examine the hypothesized two-factor structure of the PES-JP. The model showed mixed fit indices: χ^2^(19) = 127.28, *P* < 0.001, CFI = 0.949, TLI = 0.925, RMSEA = 0.120 (90% confidence interval [CI], 0.100–0.140), SRMR = 0.061 (Figure 2). Factor loadings for the approach dimension ranged from 0.82 to 0.90 (standardized loadings), while loadings for the avoidance dimension ranged from 0.76 to 0.84 (standardized loadings). The estimate of correlation between the approach and avoidance factors was *r* = −0.51, *P* < 0.001. Both subscales demonstrated high internal consistency. Cronbach’s alpha was 0.92 for the approach dimension and 0.87 for the avoidance dimension, indicating excellent reliability for both subscales.

**Figure 2.**
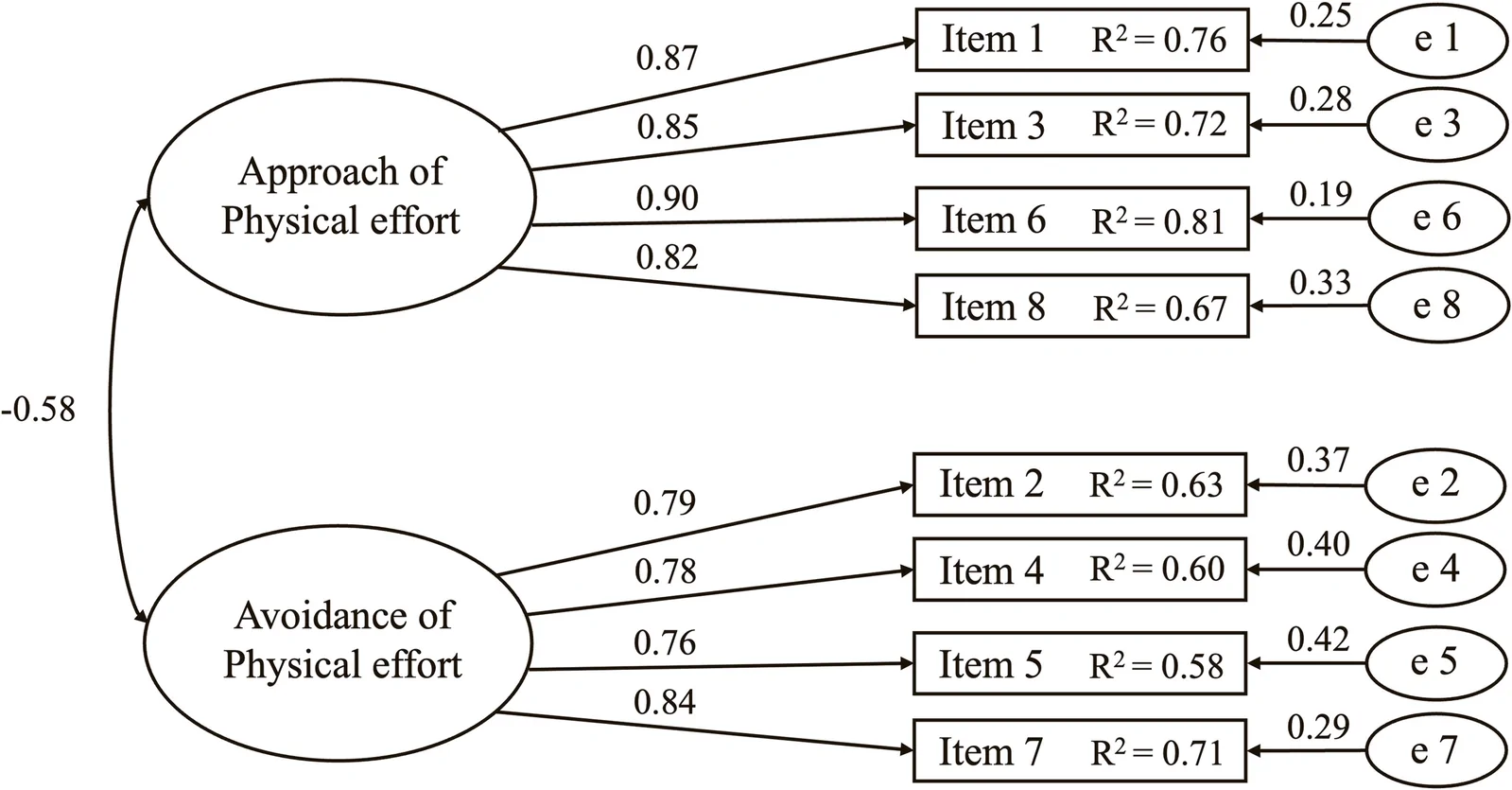
Results of the confirmatory factor analysis of the 8-item physical effort scale (PES). Standardized factor loadings are presented. e, error variance; R2, percentage of variance explained.

### Concurrent validity

Concurrent validity was assessed by examining the associations between the approach dimension, avoidance dimension, and global score of the PES-JP and PA levels as measured by the IPAQ-SF, with each score tested in a separate model (Table 1). The approach dimension of the PES-JP was associated with MVPA, moderate PA, and vigorous PA and sitting time. The avoidance dimension of the PES-JP was not associated with PA levels but was associated with sitting time. The global score was associated with MVPA, vigorous PA, and sitting time, but not with moderate PA. These results were further supported by hierarchical regression analyses that examined the associations between PES-JP scores and both MVPA and sitting time, controlling for age and sex (Table 2 and Table 3). Specifically, for MVPA, the approach dimension (β = 0.19, *P* < 0.001) and the global score (β = 0.19, *P* < 0.001) showed significant positive associations, while the avoidance dimension (β = −0.13, *P* = 0.009) showed a significant negative association. For sitting time, all PES-JP scores were significantly associated: the approach dimension (β = −0.17, *P* < 0.001) and global score (β = −0.22, *P* < 0.001) were negatively associated, whereas the avoidance dimension showed a positive association (β = 0.20, *P* < 0.001). Age did not significantly influence the observed associations between PES-JP dimensions and PA behaviors.

**Table 1.** Concurrent validity of the PES-JP approach, avoidance, and global scores

	Approach of physical effort			Avoidance of physical effort			Global of physical effort		
		
	*N*	β	*P*	*N*	β	*P*	*N*	β	*P*
MVPA	398	0.19	**<0.001**	398	−0.13	**0.008**	398	0.19	**<0.001**
Moderate PA	398	0.17	**0.001**	398	−0.02	0.679	398	0.11	**0.024**
Vigorous PA	398	0.16	**0.001**	398	−0.16	**0.001**	398	0.19	**<0.001**
Walking	398	0.08	0.116	398	−0.06	0.215	398	0.08	0.104
Sitting	398	−0.17	**<0.001**	398	0.20	**<0.001**	398	−0.22	**<0.001**

MVPA, moderate-to-vigorous physical activity; PA, physical activity; PES-JP, Japanese version of the Physical Effort Scale.

Usual levels of PA and MVPA as assessed using the International Physical Activity Questionnaire-Short Form (IPAQ-Short Form; Craig et al, 2003); Univariate linear regressions were used to assess the associations. All the variables were scaled to obtain standardized coefficients.

**Table 2.** Results of the hierarchical regression analyses for explaining the usual level of MVPA

	Baseline		Approach		Avoidance		Global	
			
	β	*P*	β	*P*	β	*P*	β	*P*
**Dependent variable: MVPA**								
Step 1								
Age	−0.05	0.290	−0.04	0.403	−0.05	0.307	−0.04	0.367
Sex (ref. women)								
Men	0.08	0.426	0.02	0.852	0.07	0.500	0.04	0.725
Step 2								
Approach			0.19	**<0.001**				
Avoidance					−0.13	**0.009**		
Global							0.19	**<0.001**
*R* ^2^								
Adjusted *R*^2^	−0.001		0.032			0.014		0.031

MVPA, moderate-to-vigorous physical activity.

Multiple linear regressions were used to assess the associations. All the variables were scaled to obtain standardized coefficients.

**Table 3.** Results of the hierarchical regression analyses for explaining usual level of time spent sitting

	Baseline		Approach		Avoidance		Global	
			
	β	*P*	β	*P*	β	*P*	β	*P*
**Dependent variable: Usual sitting time**								
Step 1								
Age	0.06	0.240	0.05	0.335	0.06	0.259	0.05	0.319
Sex (ref. women)								
Men	0.08	0.415	0.14	0.160	0.10	0.303	0.14	0.169
Step 2								
Approach			−0.18	**<0.001**				
Avoidance					0.20	**<0.001**		
Global							−0.22	**<0.001**
*R* ^2^								
Adjusted *R*^2^	0.000		0.031		0.040		0.047	

Multiple linear regressions were used to assess the associations. All the variables were scaled to obtain standardized coefficients.

### Test-retest reliability

The test-retest reliability analysis demonstrated satisfactory stability for the PES-JP over a one-week period (*n* = 207). Weighted kappa coefficients for individual items ranged from 0.43 to 0.60, with all coefficients being statistically significant (*P* < 0.001). For the approach dimension items, kappa values ranged from 0.48 to 0.61, while for the avoidance dimension items, they ranged from 0.44 to 0.53. For the subscale scores, intraclass correlation coefficients were 0.78 (95% CI, 0.71–0.82) for the approach dimension, 0.65 (95% CI, 0.56–0.72) for the avoidance dimension, and 0.81 (95% CI, 0.76–0.85) for the global dimension.

## DISCUSSION

The aim of the present study was to develop and validate the PES-JP to assess individual tendencies toward physical effort in relation to PA behavior. The findings suggest that the PES-JP demonstrates sound psychometric properties, including high internal consistency, concurrent associations with PA behavior, and test-retests reliability.

Consistent with the original validation study,^11^ exploratory factor analysis supported a two-factor structure of the PES-JP, reflecting approach and avoidance tendencies toward physical effort. Confirmatory factor analysis further examined this hypothesized structure, but several model fit indices did not fully meet conventional criteria, suggesting limited overall model fit. Nevertheless, the approach and avoidance subscales were moderately negatively correlated, which is a desirable pattern, as it indicates that the two dimensions capture distinct yet related constructs and supports the relevance of considering a two-factor solution. In addition, factor loadings were strong and well differentiated, and residual-level fit was acceptable. Accordingly, the proposed factor structure should be considered preliminary and interpreted with appropriate caution. Importantly, both subscales demonstrated high internal consistency, indicating reliable measurement of approach and avoidance tendencies. The magnitude of the negative association between the approach and avoidance subscales was somewhat lower than that reported in the original study, which may reflect cultural or linguistic differences in how individuals conceptualize and respond to approach versus avoidance of physical effort. Overall, while further research is needed to more rigorously establish the structural validity of the PES-JP, the present findings provide a reasonable basis for its cautious use and continued psychometric evaluation.

In terms of concurrent validity, the approach dimension and global score were positively associated with MVPA and negatively associated with sitting time. Conversely, the avoidance dimension showed a negative association with MVPA and a positive association with sitting time. These results mirror the original findings,^11^ suggesting that individuals with a stronger tendency to approach physical effort are more likely to engage in PA, whereas those with a stronger tendency to avoid effort may be more inclined toward sedentary behavior. This pattern remained robust in hierarchical regression analyses controlling for age and sex, underscoring the importance of assessing effort-related tendencies in understanding PA behavior. In sum, the PES-JP reproduced the concurrent validity pattern reported in the original study, demonstrating that the scale can be used with Japanese populations and across a wider age range than the original version. Taken together, the PES-JP provides a useful tool for investigating how individual differences in tendencies toward physical effort are associated with PA behavior, and for advancing research on effort-related mechanisms underlying everyday PA across cultural contexts.

Regarding reliability, the PES-JP showed adequate temporal stability, with both item-level agreement and subscale-level consistency falling within acceptable ranges. These findings suggest that individual tendencies toward physical effort, as assessed by the PES-JP, are relatively stable over short periods of time. This supports the scale’s utility in longitudinal or repeated-measures research designs, where consistency over time is essential.

Several strengths of this study should be noted. First, it included a relatively large sample size covering a wide age range of Japanese adults. Second, the PES was translated following rigorous cross-cultural adaptation guidelines, including back-translation and cognitive debriefing procedures. Third, the study benefited from the involvement of one of the original developers of the PES as a co-author (BC), which ensured conceptual fidelity and strengthened the cross-cultural adaptation process. These methodological strengths provide confidence in the robustness of the findings. Nevertheless, several limitations should also be acknowledged. First, although exploratory analyses supported a two-factor structure of the PES-JP, confirmatory factor analysis yielded mixed model fit indices. Thus, the structural validity of the PES-JP should be regarded as preliminary and warrants further examination in larger and independent samples. Second, the reliance on self-reported PA measures may introduce recall bias. Self-reports tend to overestimate PA levels and underestimate sitting time when compared to objective measures, such as accelerometry,^23^^,^^24^ potentially leading to inflated associations or misclassification of activity patterns. Future studies could incorporate device-based PA measures to validate and extend the present findings. Third, although our sample included participants across a wide age range and both sexes, it may not fully represent other population segments, such as older adults or clinical populations. Additional studies are needed to confirm the generalizability of the PES-JP in diverse groups. Finally, while the cross-sectional design enabled a robust psychometric validation, longitudinal or intervention studies are warranted to investigate the causal relationships between effort tendencies and PA behavior.

In summary, the PES-JP demonstrates sound psychometric properties and provides an appropriate tool for assessing tendencies to approach or avoid physical effort. The scale contributes to a better understanding of how dispositions toward physical effort relate to PA behavior. In addition, the PES-JP may have several practical measurement applications. For example, the scale can be used to identify individuals who may be more likely to disengage from effortful physical activity. This information may help inform considerations regarding how training or exercise sessions are structured or implemented, such as decisions related to pacing, framing of effort, and instructional strategies. The PES-JP can also be used as a measurement indicator to examine how interventions, such as affective or experiential approaches, are related to individuals’ evaluation of physical effort. In this context, changes in PES-JP scores should be interpreted as reflecting shifts in effort valuation rather than changes in affect per se. Beyond these applications, the PES-JP can contribute to cross-cultural research by capturing individual differences in dispositions toward physical effort. Future studies should further explore these associations using device-based PA indicators and research designs that can clarify causal relationships.

## SUPPLEMENTARY MATERIAL

eMaterial 1. Supplemental methods

eMaterial 2. A preliminary validation study of PES-JP item 5 with and without explanatory labels

eMaterial 3. Associations between PES-JP and selected psychological constructs

eMaterial 4. Distribution of physical activity

eMaterial 5. Japanese version of the Physical Effort Scale

eTable 1. Concurrent, convergent, and discriminant validity of PES-JP Item 5 across two versions (with and without explanatory labels)

eTable 2. Associations of PES-JP approach, avoidance, and global scores with selected psychological constructs

eFigure 1. Correlation between the approach and avoidance dimension of physical effort and the other assessed variables. Correlation coefficients are represented as colored circles, with the bluer and larger circles indicating the coefficients were closer to +1, the redder and larger circles indicating the coefficients were closer to −1, and the whiter and smaller circles indicating a coefficient closer to 0.

eFigure 2. Physical activity distribution in the preliminary validation study. MET, metabolic equivalent of task.

## References

[1] Kohl HW 3rd, Craig CL, Lambert EV, ; Lancet Physical Activity Series Working Group. The pandemic of physical inactivity: global action for public health. Lancet. 2012;380(9838):294–305. 10.1016/S0140-6736(12)60898-8 22818941

[2] Lee IM, Shiroma EJ, Lobelo F, ; Lancet Physical Activity Series Working Group. Effect of physical inactivity on major non-communicable diseases worldwide: an analysis of burden of disease and life expectancy. Lancet. 2012;380(9838):219–229. 10.1016/S0140-6736(12)61031-9 22818936 PMC3645500

[3] Rhodes RE, McEwan D, Rebar AL. Theories of physical activity behaviour change: a history and synthesis of approaches. Psychol Sport Exerc. 2019;42:100–109. 10.1016/j.psychsport.2018.11.010

[4] Zhang CQ, Zhang R, Schwarzer R, Hagger MS. A meta-analysis of the health action process approach. Health Psychol. 2019;38(7):623–637. 10.1037/hea0000728 30973747

[5] Schwarzer R. Modeling health behavior change: how to predict and modify the adoption and maintenance of health behaviors. Appl Psychol. 2008;57(1):1–29. 10.1111/j.1464-0597.2007.00325.x

[6] Cheval B, Boisgontier MP. The theory of effort minimization in physical activity. Exerc Sport Sci Rev. 2021;49(3):168–178. 10.1249/JES.0000000000000252 34112744 PMC8191473

[7] Bellettiere J, Nguyen B, Liles S, . Prompts to increase physical activity at points-of-choice between stairs and escalators: what about escalator climbers? Transl Behav Med. 2019;9(4):656–662. 10.1093/tbm/iby080 30099542 PMC6629843

[8] Maltagliati S, Fessler L, Yu Q, . Effort minimization: a permanent, dynamic, and surmountable influence on physical activity. J Sport Health Sci. 2025;14(100971):100971. 10.1016/j.jshs.2024.100971 39233203 PMC11809139

[9] Bustamante LA, Oshinowo T, Lee JR, . Effort Foraging Task reveals positive correlation between individual differences in the cost of cognitive and physical effort in humans. Proc Natl Acad Sci USA. 2023;120(50):e2221510120. 10.1073/pnas.2221510120 38064507 PMC10723129

[10] Treadway MT, Buckholtz JW, Cowan RL, . Dopaminergic mechanisms of individual differences in human effort-based decision-making. J Neurosci. 2012;32(18):6170–6176. 10.1523/JNEUROSCI.6459-11.2012 22553023 PMC3391699

[11] Cheval B, Maltagliati S, Courvoisier DS, Marcora S, Boisgontier MP. Development and validation of the physical effort scale (PES). Psychol Sport Exerc. 2024;72(102607):102607. 10.1016/j.psychsport.2024.102607 38364989

[12] Craig CL, Marshall AL, Sjöström M, . International physical activity questionnaire: 12-country reliability and validity. Med Sci Sports Exerc. 2003;35(8):1381–1395. 10.1249/01.MSS.0000078924.61453.FB 12900694

[13] St-Denis B, Beaudry S, Boisgontier MP, Cheval B, Maltagliati S. Validation de la version francophone de l’Échelle d’Effort Physique. Mov Sport Sci. 2024;126:33–45. 10.1051/sm/2024019

[14] Wild D, Grove A, Martin M, ; ISPOR Task Force for Translation and Cultural Adaptation. Principles of Good Practice for the translation and Cultural Adaptation process for patient-reported outcomes (PRO) measures: Report of the ISPOR task force for translation and Cultural Adaptation. Value Health. 2005;8(2):94–104. 10.1111/j.1524-4733.2005.04054.x 15804318

[15] Cross Marketing Inc. https://www.cross-m.co.jp/en/. Accessed 30.09.2025.

[16] Terwee CB, Bot SDM, de Boer MR, . Quality criteria were proposed for measurement properties of health status questionnaires. J Clin Epidemiol. 2007;60(1):34–42. 10.1016/j.jclinepi.2006.03.012 17161752

[17] Murase N, Katsumura T, Ueda C, Inoue S, Shimomitsu T. Validity and reliability of Japanese version of International Physical Activity Questionnaire. J Health Welfare Stat. 2002;49(11):1–9.

[18] Hu LT, Bentler PM. Cutoff criteria for fit indexes in covariance structure analysis: conventional criteria versus new alternatives. Struct Equ Modeling. 1999;6(1):1–55. 10.1080/10705519909540118

[19] Shi D, Maydeu-Olivares A, Rosseel Y. Assessing fit in ordinal factor analysis models: SRMR vs. RMSEA. Struct Equ Modeling. 2020;27(1):1–15. 10.1080/10705511.2019.1611434

[20] McHugh ML. Interrater reliability: the kappa statistic. Biochem Med (Zagreb). 2012;22(3):276–282. 10.11613/BM.2012.031 23092060 PMC3900052

[21] Koo TK, Li MY. A guideline of selecting and reporting intraclass correlation coefficients for reliability research. J Chiropr Med. 2016;15(2):155–163. 10.1016/j.jcm.2016.02.012 27330520 PMC4913118

[22] Scoring protocol for the International Physical Activity Questionnaire. https://sites.google.com/view/ipaq/score. Accessed 07.11.2025.

[23] Dyrstad SM, Hansen BH, Holme IM, Anderssen SA. Comparison of self-reported versus accelerometer-measured physical activity. Med Sci Sports Exerc. 2014;46(1):99–106. 10.1249/MSS.0b013e3182a0595f 23793232

[24] Gardner B, Flint S, Rebar AL, . Is sitting invisible? Exploring how people mentally represent sitting. Int J Behav Nutr Phys Act. 2019;16(1):85. 10.1186/s12966-019-0851-0 31606040 PMC6790031

