# Supplementary material for: Development and Psychometric Evaluation of the Japanese Version of the Physical Effort Scale
Source: J Epidemiol. 2026 Oct 5;36(10):321–7. doi: 10.2188/jea.JE20250530 (PMC13562595; doi:10.2188/jea.JE20250530)
Supplement: Supplementary file 1 [file je-36-321-s001.zip › JE20250530_eMaterial_eTable_eFigure_accepted_36-10-clean_EO.pdf]

**eMaterial 1.** Supplemental methods

## Scale translation

The translation process followed the guidelines of Wild et al.[1] The procedural flow of the scale translation is illustrated in **Figure 1**. Two Japanese authors (KS and KK), with expertise in sport/exercise psychology, independently translated the English version of the PES into Japanese. Subsequently, these two authors, along with another Japanese author (HS), who has expertise in the assessment of physical activity (PA), discussed the cultural context and appropriateness of the forward-translated version, and then collaborated to create a consensus Japanese version. Next, a professional bilingual translator from an English editing company specializing in academic manuscripts, who was kept unaware of the underlying concepts of the Physical Effort Scale (PES), back-translated the Japanese consensus version into English. The three authors who created the Japanese consensus version reviewed the back-translated items and found no issues. The developer of the original version (BC) reviewed the back-translated items and confirmed their conceptual equivalence with the original version. Based on this confirmation, the Japanese consensus version was adopted as the pre-final version. Subsequently, cognitive briefing was conducted with eight Japanese undergraduate and

graduate students (mean age, 23.9 years; 4 females) to assess the comprehensibility and cognitive equivalence of the pre-final Japanese version of the PES. Based on the feedback obtained, the Japanese authors discussed the findings and made minor revisions to enhance clarity and consistency. In particular, Item 5 (exerting physical effort does not appeal to me) was revised in response to feedback noting that, due to its negative phrasing, it required reverse interpretation of the 5-point Likert scale, which could cause confusion regarding the scale direction. Such negatively worded items are particularly prone to misunderstanding in Japanese due to linguistic differences between English and Japanese. To address this issue, we created two versions of the item: one with brief explanatory labels added to each scale point (eg, “strongly appealing” to “not appealing at all”), and one without such labels. A preliminary validation study involving 200 participants was conducted to examine which version demonstrated better construct validity. The results indicated that the version with explanatory labels demonstrated stronger concurrent, convergent, and discriminant validity than the version without them (eMaterial 2). Therefore, we designated the version including the explanatory labels as the final PES-JP. The final version is available in eMaterial 5.

**Procedure**

This study was approved by the Human Research Ethics Committee of Chukyo University (2024-016) and the Ethics Committee of the Graduate School of Medicine, Tohoku University (2025-1-190). The main survey was conducted online. The entire web-based survey process, including participant recruitment and data collection, was conducted by Cross Marketing Inc., a leading survey company in Japan with a panel of over 10 million registered monitors.[2] Cross Marketing Inc. was selected based on their proven track record in academic research, including contributions to numerous peer-reviewed publications in health and behavioral sciences (eg, Yamada et al.[3]), and their capability to execute stratified sampling procedures required for this study. Data collection was conducted in April and May 2025. A total of 400 participants were recruited for this study to ensure sufficient representation across different age groups and sexes. This sample size was determined based on commonly accepted recommendations for validation studies, which suggest recruiting at least 10 participants per item when evaluating the structure and reliability of a scale.[4] Additionally, while the original validation study of the PES by Cheval et al.[5] (study 3) included 297 undergraduate students, we selected a larger and more diverse sample spanning ages 20–59 years to enhance the generalizability of the findings. Prior to accessing the questionnaires, all participants were given a detailed explanation of the study and

provided their informed consent electronically. To be eligible for the study, participants had to meet the following criteria: 1) be native Japanese speakers; 2) be able to engage in PA without any medical contraindications; and 3) be aged between 20 and 59 years. In the preliminary validation study described above, we observed a relatively high proportion of participants reporting that they did not engage in any PA (eMaterial 4). Although the exact reason for this finding remains unclear, it may be partly attributable to the nature of online surveys. For instance, individuals with more sedentary lifestyles may be more likely to register as monitors for web-based research panels. To address this issue, we consulted data from a nationally representative survey conducted in Japan,[6] which reported that 26% of adults aged 20–59 years did not engage in any PA. Accordingly, participant recruitment was stratified to reflect this distribution. Specifically, within each sex-by-age group stratum (ie, 20s, 30s, 40s, and 50s; 50 participants per stratum), 13 individuals (26%) were purposively sampled to represent inactive individuals.

## Measures

### *PES-JP*

Participants were instructed to complete the PES-JP by responding to eight statements

using a five-point Likert scale ranging from 1 (“I completely disagree”) to 5 (“I completely agree”). Four items assessed the tendency to approach physical effort, and four items assessed the tendency to avoid it. Consistent with the initial validation study,[5] a global score was calculated to reflect the tendency to approach (rather than avoid) physical effort. The global score was computed by subtracting the mean avoidance tendency score from the mean approach tendency score (Global score = Approach − Avoidance). This score theoretically ranges from −4 to 4, with higher scores indicating a stronger tendency to approach physical effort and lower scores indicating a greater tendency to avoid it.

***Concurrent validity measurements***

Based on the original study[5] and French versions of the PES,[7] PA levels were assessed to examine the concurrent validity using a Japanese version of the International Physical Activity Questionnaire - Short Form (IPAQ-SF).[8] The IPAQ-SF enables the measurement of overall PA in a typical week, accounting for various intensities and types of PA. Participants were instructed to report both the number of days per week and the total duration per day (in hours and minutes) for vigorous-intensity PA, moderate-intensity PA, and walking performed in bouts of at least 10 consecutive

minutes, as well as the time spent in sedentary behaviors, including sitting and reclining, in a typical week. PA levels were calculated as MET-minutes per week, according to the standard IPAQ scoring protocol.[9] MVPA was determined as the sum of MET-minutes per week from the vigorous-intensity, moderate-intensity, and walking categories. Following the IPAQ scoring guidelines,[9] cases in which the total time of all walking, moderate, and vigorous activities exceeded 960 minutes per day were excluded from the analysis.

***Psychological constructs referenced in prior PES research***

Following the procedures and theoretical rationale described in the original and French development studies,[5, 7] the psychological constructs listed below were included and analyzed for the purpose of comparison with the original and French versions of the PES, in order to illustrate the conceptual framework underlying the Theory of Effort Minimization in Physical Activity.[10]

***Autonomous and controlled motivation***[11]

Autonomous motivation refers to engaging in PA because it is personally valued, enjoyable, or aligned with one's interests and sense of self. It reflects a sense of volition

and personal endorsement of the behavior, rather than external pressure. Controlled motivation refers to engaging in PA due to internal or external pressure, such as feelings of obligation, guilt, or external demands and rewards. It reflects a more coercive form of motivation, in which behavior is regulated by pressure rather than personal choice.

### *Automaticity*[12]

Automaticity refers to the extent to which a behavior is performed without conscious awareness, deliberation, or intentional control, rather than deliberate decision-making.

### *Affective and instrumental attitudes*[13]

Affective attitude refers to the experiential or emotional evaluation of engaging in PA, such as whether performing the behavior is perceived as pleasant, enjoyable, or unpleasant. Instrumental attitude refers to the cognitive or utilitarian evaluation of engaging in PA, such as whether the behavior is perceived as beneficial, valuable, or useful for achieving desired outcomes.

### *Intention*[14]

Intention refers to an individual's conscious plan or stated readiness to engage in PA in

the future. It reflects a deliberate motivational commitment to perform the behavior, representing the proximal antecedent of action in many social–cognitive models of PA.

Prior theoretical and empirical work has consistently shown positive associations between PA behavior and autonomous motivation, affective attitudes, intentions, and automaticity.[15–17] Within this literature, tendencies to approach physical effort have typically been discussed in relation to higher levels of these constructs, whereas tendencies to avoid physical effort have been discussed in relation to lower levels. Based on this background, the original and French PES development studies[5, 7] referenced these constructs to situate effort-related tendencies within broader theoretical frameworks of PA behavior. Accordingly, individuals with a higher tendency to approach physical effort were expected to display higher levels of autonomous motivation, affective attitudes, and intentions, whereas individuals with a stronger tendency to avoid physical effort were expected to show the opposite pattern.

In contrast, constructs such as controlled motivation and instrumental attitudes are conceptually distinct from automatic effort-related tendencies. Rather, they have been discussed as reflecting more deliberative motivational or cognitive influences on PA behavior.[15] This conceptual distinction, emphasized in the original PES validation study,[5] highlights how tendencies toward physical effort are differentiated from more

reflective forms of motivation or evaluation. Consistent with this conceptual distinction, controlled motivation and instrumental attitudes were expected to show only moderate associations with the PES-JP (both approach and avoidance dimensions), as these constructs reflect more conscious, goal-directed motivational or evaluative processes rather than automatic effort-related tendencies.

All psychological constructs were assessed using Japanese-language questionnaires. Automaticity toward PA was measured using a validated Japanese version developed in previous research.[18] For the other constructs (motivation, attitudes, and intention), no validated Japanese versions were available; therefore, we created Japanese versions through a simplified forward-translation and expert review process. Specifically, in a manner consistent with the translation procedure used for the PES-JP, two Japanese authors (KS and KK) independently translated the original English scales into Japanese. Subsequently, these two authors, together with another Japanese author (HS), discussed the linguistic accuracy and cultural appropriateness of the translations and finalized the Japanese versions after reaching full agreement. To ensure measurement reliability, Cronbach's α coefficients for these scales are reported in eMaterial 3.

Associations between PES-JP scale scores and selected psychological

constructs were examined using regression analyses for descriptive purposes. Specifically, relationships with autonomous motivation, affective attitudes, intention, automaticity, as well as controlled motivation and instrumental attitudes were examined to situate PES-JP scores within the theoretical context discussed in prior PES research.[5, 7] These constructs have been referenced to illustrate how effort-related tendencies relate to both more automatic and more reflective motivational or evaluative processes.

***Test-retest reliability***

To assess the test-retest reliability of the PES-JP, a subsample of participants who completed the initial online questionnaire were asked to complete the PES-JP again after a 7-day interval, following the procedure used in the original version.[5] The procedure for the second administration of the PES-JP was identical to that of the first, ensuring consistency in data collection methods.

**REFERENCES**

1. Wild D, Grove A, Martin M, et al. ISPOR Task Force for Translation and Cultural Adaptation. Principles of Good Practice for the translation and Cultural Adaptation process for patient-reported outcomes (PRO) measures: Report of the ISPOR task force for translation and Cultural Adaptation. *Value Health*. 2005;8(2):94-104. doi:10.1111/j.1524-4733.2005.04054.x

2. Cross Marketing Inc. https://www.cross-m.co.jp/en/

3. Yamada Y, Namba H, Date H, et al. Regional difference in the impact of COVID-19 pandemic on domain-specific physical activity, sedentary behavior, sleeping time, and step count: Web-based cross-sectional nationwide survey and accelerometer-based observational study. *JMIR Public Health Surveill*. 2023;9(1):e39992. doi:10.2196/39992

4. Terwee CB, Bot SDM, de Boer MR, et al. Quality criteria were proposed for measurement properties of health status questionnaires. *J Clin Epidemiol*. 2007;60(1):34-42. doi:10.1016/j.jclinepi.2006.03.012

5. Cheval B, Maltagliati S, Courvoisier DS, Marcora S, Boisgontier MP. Development and validation of the physical effort scale (PES). *Psychol Sport*

*Exerc*. 2024;72(102607):102607. doi:10.1016/j.psychsport.2024.102607

6. Sasakawa Sports Foundation. https://www.ssf.or.jp/thinktank/sports_life/datalist/2022/index.html

7. St-Denis B, Beaudry S, Boisgontier MP, Cheval B, Maltagliati S. Validation de la version francophone de l'Échelle d'Effort Physique. *Mov Sport Sci*. 2024;(126):33-45. doi:10.1051/sm/2024019

8. Murase N, Katsumura T, Ueda C, Inoue S, Shimomitsu T. Validity and reliability of Japanese version of International Physical Activity Questionnaire. *Journal of Health and Welfare Statistics*. 2002;49(11):1-9.

9. Scoring protocol for the International Physical Activity Questionnaire. https://sites.google.com/view/ipaq/score

10. Cheval B, Boisgontier MP. The theory of effort minimization in physical activity. *Exerc Sport Sci Rev*. 2021;49(3):168-178. doi:10.1249/JES.0000000000000252

11. Brunet J, Gunnell KE, Gaudreau P, Sabiston CM. An integrative analytical framework for understanding the effects of autonomous and controlled motivation. *Pers Individ Dif*. 2015;84:2-15. doi:10.1016/j.paid.2015.02.034

12. Gardner B, Abraham C, Lally P, de Bruijn GJ. Towards parsimony in habit measurement: testing the convergent and predictive validity of an automaticity subscale of the Self-Report Habit Index. *Int J Behav Nutr Phys Act*. 2012;9(1):102. doi:10.1186/1479-5868-9-102

13. Ajzen I. Constructing a TPB questionnaire: Conceptual and methodological considerations. Published online 2002. https://citeseerx.ist.psu.edu/document?repid=rep1&type=pdf&doi=0574b20bd58130dd5a961f1a2db10fd1fcbae95d

14. Rhodes RE, Rebar AL. Conceptualizing and defining the intention construct for future physical activity research. *Exerc Sport Sci Rev*. 2017;45(4):209-216. doi:10.1249/JES.0000000000000127

15. Hagger MS, Chatzisarantis NLD, Biddle SJH. The influence of autonomous and controlling motives on physical activity intentions within the Theory of Planned Behaviour. *Br J Health Psychol*. 2002;7(Part 3):283-297. doi:10.1348/135910702760213689

16. Rhodes RE, Fiala B, Conner M. A review and meta-analysis of affective judgments and physical activity in adult populations. *Ann Behav Med*. 2009;38(3):180-204.

doi:10.1007/s12160-009-9147-y

17. Maltagliati S, Sarrazin P, Isoard-Gautheur S, Pelletier L, Rocchi M, Cheval B. Automaticity mediates the association between action planning and physical activity, especially when autonomous motivation is high. *Psychol Health*. 2025;40(1):67-83. doi:10.1080/08870446.2023.2188886

18. Takami K. Development of Japanese version of the self-report habit index. *Bulletin of the Graduate School of Human Development and Environment, Kobe University*. 2023;(2):29-39. doi:10.24546/0100481767 [in Japanese]

**eMaterial 2.** A preliminary validation study of PES-JP item 5 with and without explanatory labels

**Study Overview**

A preliminary validation study involving 200 participants was conducted to examine whether the version of Physical Effort Scale – Japanese version (PES-JP) Item 5 (exerting physical effort does not appeal to me) with explanatory labels on the scale points demonstrated better construct validity than the version without such labels. The results indicated that the labeled version showed stronger concurrent, convergent, and discriminant validity. According to the International Physical Activity Questionnaire (IPAQ) scoring criteria, one participant reporting a total physical activity (PA) time exceeding 960 minutes per day was excluded from the analysis.

**Group classification**

Group 1 (n=99): Participants who completed the questionnaire version with explanatory labels added to the scale points of Item 5.

Group 2 (n=100): Participants who completed the version without explanatory labels.

**Concurrent validity**

Concurrent validity was assessed by examining the associations between PES-JP Item 5 and PA levels as measured using the IPAQ, comparing responses between Group 1 (with explanatory labels) and Group 2 (without explanatory labels). The results are summarized in eTable 1.

In Group 1, Item 5 showed significant trend associations with vigorous PA (β= –0.20, *P*=0.053), walking (β= –0.25, *P*=0.013), and sitting time (β=0.22, *P*=0.026). No significant associations were found with moderate PA or moderate-to-vigorous PA (MVPA).

In Group 2, the version without explanatory labels showed weaker concurrent validity. Item 5 was significantly associated only with sitting time (β =0.20, *P*=0.045), with no significant associations observed for MVPA, moderate PA, vigorous PA, or walking.

Based on these findings, the version with explanatory labels was selected for Item 5 in the main analyses, given its more consistent pattern of associations with PA-related measures.

**eTable 1.** Concurrent, convergent, and discriminant validity of PES-JP Item 5 across two versions (with and without explanatory labels)

| | Group 1 | | | Group 2 | | |
|---|---|---|---|---|---|---|
| | N | β | *P* | N | β | *P* |
| **Concurrent validity** | 99 | | | 100 | | |
| Usual level of PA | 99 | | | 100 | | |
| MVPA | 99 | -0.18 | 0.073 | 100 | 0.00 | 0.977 |
| Moderate PA | 99 | -0.07 | 0.482 | 100 | 0.06 | 0.560 |
| Vigorous PA | 99 | -0.20 | 0.053 | 100 | -0.04 | 0.710 |
| Walking | 99 | -0.25 | **0.013** | 100 | 0.03 | 0.735 |
| Sitting | 99 | 0.22 | **0.026** | 100 | 0.20 | **0.045** |

MVPA, moderate-to-vigorous physical activity; PA, physical activity; PES-JP, Japanese version of the Physical Effort Scale.

Usual level of PA as assessed by the International Physical Activity Questionnaire-Short Form (IPAQ-Short Form; Craig et al., 2003);. Univariate linear regressions were used to assess the associations. All the variables were scaled to obtain standardized coefficients. Group 1 refers to participants who completed the version of the questionnaire with explanatory labels on Item 5 scale points, while Group 2 refers to those who completed the version without explanatory labels. Group 1 (n=99): Mean age, 41.0 (standard deviation [SD], 11.3) years, 50.5% women. Group 2 (n=100): Mean age, 40.8 (SD, 11.1) years, 50.0% women.

**eMaterial 3.** Associations between PES-JP and selected psychological constructs

The following analyses are presented for descriptive and comparative purposes only, to facilitate comparison with the original and French versions of the Physical Effort Scale (PES).[1, 2] eFigure 1 illustrates the correlations between PES – Japanese version (PES-JP) scores (approach, avoidance, and global) and the variables used to illustrate theoretically relevant associations. The PES-JP scores were examined in relation to theoretically relevant constructs: autonomous motivation, affective attitudes, and intentions, as conceptually related constructs, and controlled motivation and instrumental attitudes, as conceptually distinct constructs (eTable 2). The observed associations were consistent with the expected theoretical pattern. The approach score (β=0.49−0.75, *Ps*<0.001) and the global score (β=0.53−0.72, *Ps*<0.001) were positively associated with autonomous motivation, affective attitudes, intentions, and automaticity, whereas the avoidance score showed negative associations with these constructs (β= -0.34 to -0.49, *Ps*<0.001). In contrast, all PES-JP scores showed statistically significant but weak associations with controlled motivation and instrumental attitudes. The approach dimension (β=0.26−0.36, *Ps*<0.001) and the global score (β=0.27−0.28, *Ps*<0.001) demonstrated weak positive associations, whereas the avoidance dimension showed weak negative associations (β= -0.12 to -0.21, *Ps*<0.019), consistent with theoretical expectations.

To ensure measurement reliability, Cronbach's α coefficients were calculated for each construct in the present sample (ie, motivation, automaticity, attitudes, and intention). The internal consistency coefficients were as follows: autonomous motivation (α=0.92), controlled motivation

(α=0.80), affective attitude (α=0.95), instrumental attitude (α=0.96), intention (α=0.97), and automaticity (α=0.95). All values exceeded the conventional criterion of 0.70, indicating acceptable to excellent internal consistency. These analyses were conducted solely to confirm internal consistency within the present sample.

The approach dimension was positively associated with autonomous motivation, affective attitudes, intentions, and automaticity toward physical activity (PA), whereas the avoidance dimension showed negative associations with these variables. This pattern aligns with the predictions of the Theory of Effort Minimization in Physical Activity,[3] which emphasizes the role of effort-related tendencies in shaping individual differences in PA-related processes. The PES-JP also showed weak correlations with controlled motivation and instrumental attitudes. Consistent with the original validation study,[1] these weak associations are in line with theoretical expectations, given the theoretical distinction between effort valuation and externally regulated or utility-based constructs.

**eTable 2.** Associations of PES-JP approach, avoidance, and global scores with selected psychological constructs

| | Approach of physical effort | | | Avoidance of physical effort | | | Global of physical effort | | |
|---|---|---|---|---|---|---|---|---|---|
| | N | β | *P* | N | β | *P* | N | β | *P* |
| Autonomous motivation | 398 | 0.75 | **<0.001** | 398 | -0.49 | **<0.001** | 398 | 0.72 | **<0.001** |
| Affective attitudes | 398 | 0.49 | **<0.001** | 398 | -0.44 | **<0.001** | 398 | 0.53 | **<0.001** |
| Intentions | 398 | 0.60 | **<0.001** | 398 | -0.34 | **<0.001** | 398 | 0.54 | **<0.001** |
| Automaticity | 398 | 0.58 | **<0.001** | 398 | -0.40 | **<0.001** | 398 | 0.57 | **<0.001** |
| Controlled motivation | 398 | 0.36 | **<0.001** | 398 | -0.12 | **0.019** | 398 | 0.27 | **<0.001** |
| Instrumental attitudes | 398 | 0.26 | **<0.001** | 398 | -0.21 | **<0.001** | 398 | 0.28 | **<0.001** |

PES-JP, Japanese version of the Physical Effort Scale.

Univariate linear regressions were used to assess the associations. All the variables were scaled to obtain standardized coefficients.

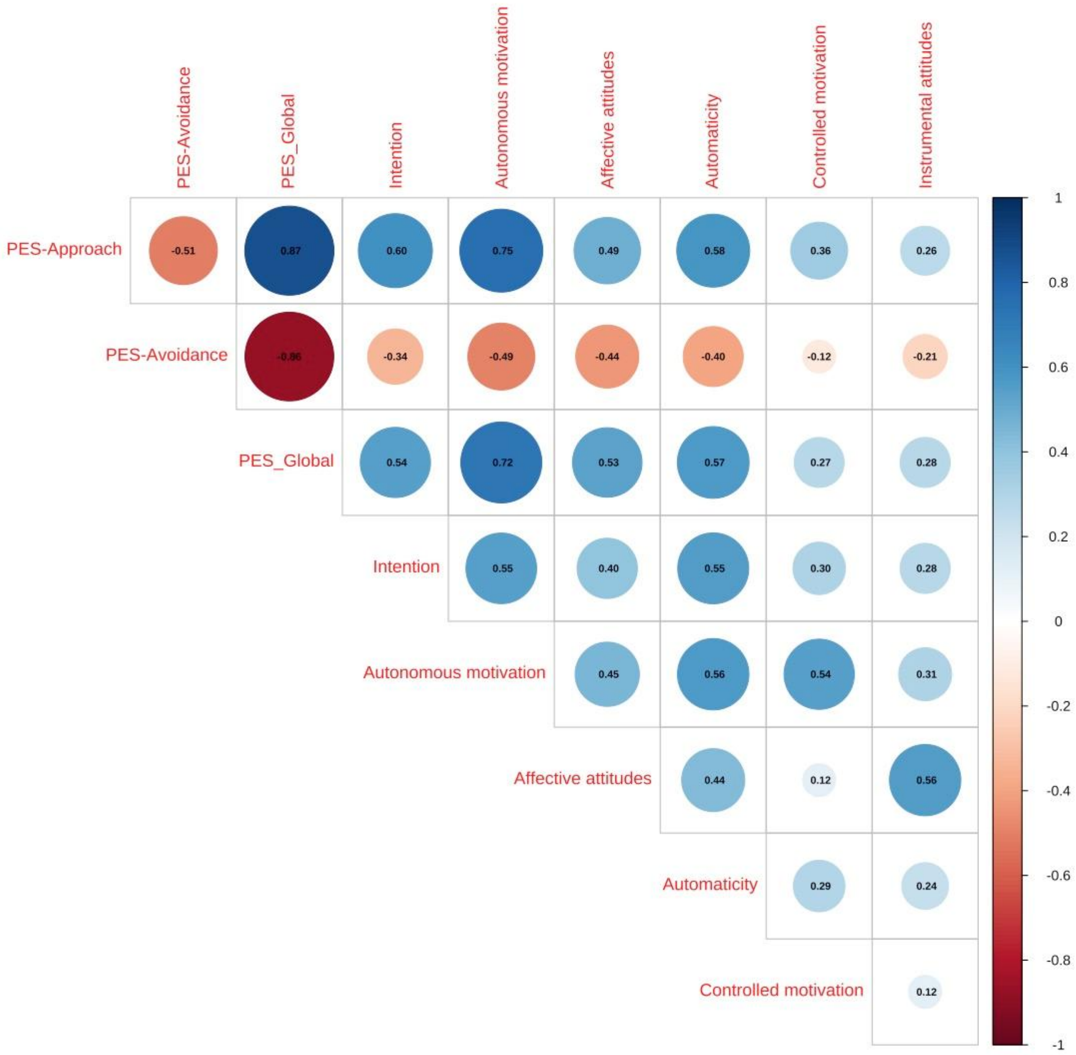


**eFigure 1.** Correlation between the approach and avoidance dimension of physical effort and the other assessed variables. Correlation coefficients are represented as colored circles, with the bluer and larger circles indicating the coefficients were closer to +1, the redder and larger circles indicating the coefficients were closer to − 1, and the whiter and smaller circles indicating a coefficient closer to 0.

**eMaterial 4.** Distribution of physical activity

A substantial proportion of participants reported no engagement in physical activity (PA) (eFigure 2). Specifically, among the 199 participants, 119 (59.8%) reported no moderate-to-vigorous PA, 142 (71.4%) reported no vigorous PA, and 140 (70.4%) reported no moderate PA. These findings underscore the high prevalence of physical inactivity in this preliminary validation study.

## REFERENCES

1. Cheval B, Maltagliati S, Courvoisier DS, Marcora S, Boisgontier MP. Development and validation of the physical effort scale (PES). *Psychol Sport Exerc*. 2024;72(102607):102607. doi:10.1016/j.psychsport.2024.102607

2. St-Denis B, Beaudry S, Boisgontier MP, Cheval B, Maltagliati S. Validation de la version francophone de l'Échelle d'Effort Physique. *Mov Sport Sci*. 2024;(126):33-45. doi:10.1051/sm/2024019

3. Cheval B, Boisgontier MP. The theory of effort minimization in physical activity. *Exerc Sport Sci Rev*. 2021;49(3):168-178. doi:10.1249/JES.0000000000000252

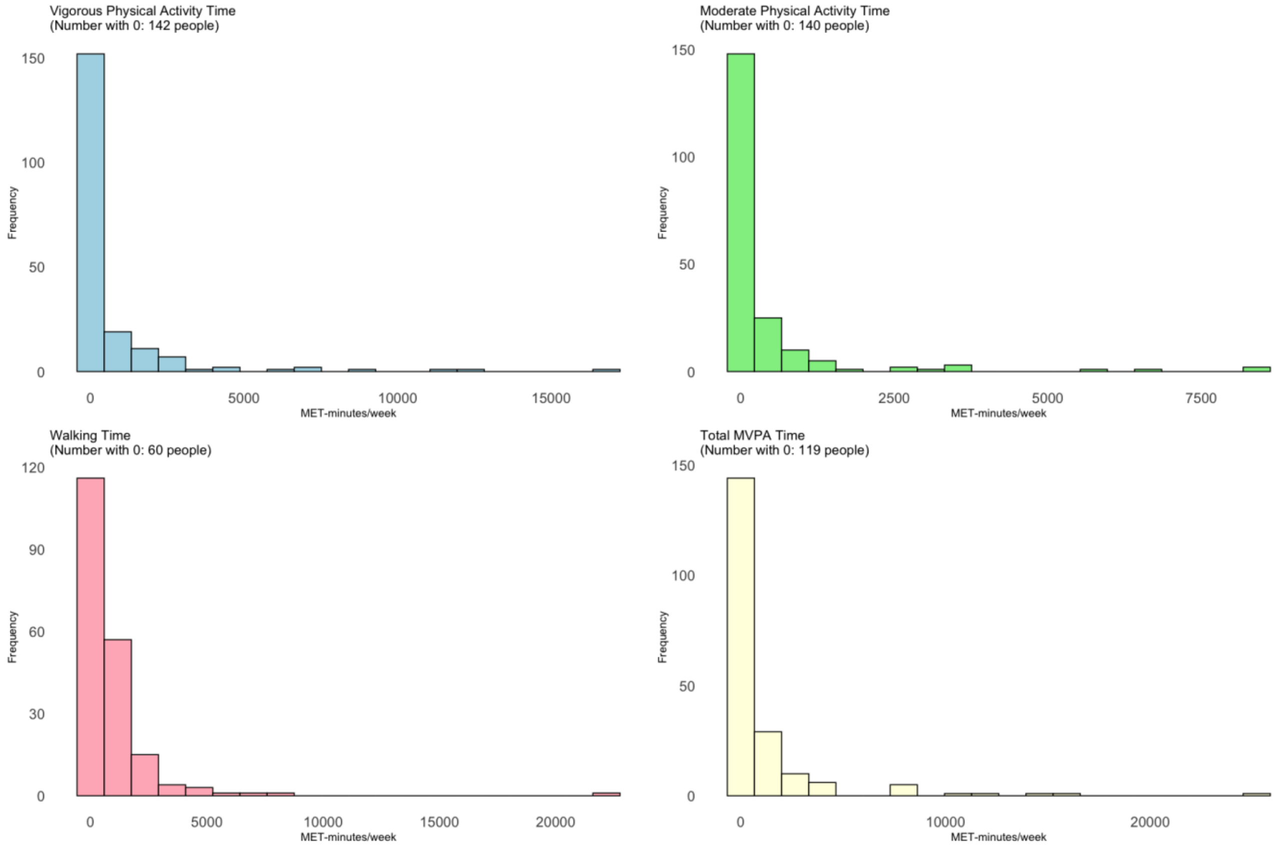


**eFigure 2.** Physical activity distribution in the preliminary validation study. MET, metabolic equivalent of task.
