# Supplementary material for: Development and Psychometric Evaluation of the Japanese Version of the Physical Effort Scale
Source: J Epidemiol. 2026 Oct 5;36(10):321–7. doi: 10.2188/jea.JE20250530 (PMC13562595; doi:10.2188/jea.JE20250530)
Supplement: Supplementary file 1 [file je-36-321-s001.zip › JE20250530-eMaterial_5-PES_JP-clean.pdf]

eMaterial 5. Japanese version of the Physical Effort Scale

Physical Effort Scale

以下の質問は、心拍数が上がったり息がはずんだりするような、身体的な努力に関するものです。**身体的な努力とは、身体活動中に自覚する労力（どのくらいがんばっているのかという感覚）のことです。**それぞれの質問について、1〜5 までの数字のうち最もよくあてはまるものに○をつけてください。回答に正解や不正解はありません。

		1 全くあてはまらない	2 あてはまらない	3 どちらでもない	4 あてはまる	5 非常によくあてはまる
1.	身体的な努力を必要とする活動が好きです。	1	2	3	4	5
2.	身体的な努力を求められる状況を避けることが多いです。	1	2	3	4	5
3.	身体的な努力を費やすことを思うと前向きな気持ちになります。	1	2	3	4	5
4.	身体的な努力を必要とする作業から距離をおく傾向があります。	1	2	3	4	5
5.	身体的な努力を費やすことに魅力を感じません。（この質問のみ否定文なので、誤解のないように選択肢に説明を加えています。）	1（非常に魅力を感じる）	2（魅力を感じる）	3（どちらでもない）	4（魅力を感じない）	5（全く魅力を感じない）
6.	身体的な努力を伴う活動は楽しいです。	1	2	3	4	5
7.	身体的な努力を伴う活動が嫌いです。	1	2	3	4	5
8.	身体的な努力を伴う活動に進んで取り組みます。	1	2	3	4	5
